# Immunohistochemistry as a Reliable Tool for the Diagnosis of Cystic Echinococcosis in Patients from Sardinia, Italy—A Confirmatory Study

**DOI:** 10.3390/diseases12050084

**Published:** 2024-04-26

**Authors:** Cinzia Santucciu, Angela Peruzzu, Antonella Maria Fara, Antonio Cossu, Philipp A. Kronenberg, Peter Deplazes, Giovanna Masala

**Affiliations:** 1 WOAH and National Reference Laboratory for Echinococcosis, Animal Health, Istituto Zooprofilattico Sperimentale della Sardegna, 07100 Sassari, Italy; angela.peruzzu@izs-sardegna.it (A.P.); giovanna.masala@izs-sardegna.it (G.M.); 2 Department of Biomedical Sciences, Institute of Pathology, University of Sassari, 07100 Sassari, Italy; antonellam.fara@tiscali.it (A.M.F.); cossu@uniss.it (A.C.); 3 Institute of Parasitology, Vetsuisse and Medical Faculty, University of Zurich, 8091 Zurich, Switzerland; philipp.kronenberg2@uzh.ch (P.A.K.); peter.deplazes@uzh.ch (P.D.); 4 Medical Micro- and Molecular Biology, Institute of Chemistry and Biotechnology, Zurich University of Applied Sciences (ZHAW), 8820 Wädenswil, Switzerland; 5 Clinics of Hepatology and Gastroenterology, University Hospital Zurich, 8091 Zurich, Switzerland

**Keywords:** cystic echinococcosis, *Echinococcus granulosus sensu stricto*, echinococcal cyst, diagnostic tool, immunohistochemistry, monoclonal antibody, mAbEm2G11, mAbEmG3

## Abstract

Cystic Echinococcosis (CE) is a zoonotic disease caused by the larval stage of the tapeworm *Echinococcus granulosus sensu lato (s.l.)*. This study aims to investigate the use of two monoclonal antibodies (mAbEmG3 and mAbEm2G11) by immunohistochemistry (IHC) to confirm the diagnosis of CE in human patients, in particular in those cases in which other techniques fail to provide a correct or conclusive diagnosis. For this purpose, a survey on 13 patients was performed. These subjects were referred to Sardinian hospitals (Italy) from 2017 to 2022 and were suspected to be affected by CE. Our findings from these 13 patients showed the detection of *E. granulosus sensu stricto* by IHC in 12 of 13 echinococcal cysts, as one sample was of a non-parasitological origin. The results confirmed that IHC, by means of the mAbEmG3 and mAbEm2G11, is a reliable diagnostic tool that showed a very high performances when tested on strain of *E. granulosus s.l.* from Sardinia.

## 1. Introduction

Echinococcosis is a zoonotic disease caused by the larval stage (metacestodes) of tapeworms of the genus *Echinococcus* belonging to Cestoda class and Taeniidae family [1,2]. In humans, the species with the most medical importance are *E. granulosus sensu lato* (*s.l.*), the causative agent of cystic echinococcosis (CE), and *E. multilocularis*, the etiological agent of alveolar echinococcosis (AE) [3]. These two helminths represent a serious health problem worldwide give different clinical symptoms and have, host specificity, pathogenicity, and geographic distributions [4,5,6,7]. 

*E. granulosus s.l.* has a cosmopolitan distribution, except for Antarctica. It has been eliminated in Iceland and New Zealand through comprehensive control programs, and is highly endemic in Mediterranean areas [8,9]. Italy presents an overall incidence rate of human CE equal to 1.6/10^5^ inhabitants per year [5,10]. Differences have been recorded among the Italian administrative divisions, the regions, with the highest incidence in the islands of Sardinia and Sicily corresponding to 6.8/10^5^ and 4.0/10^5^, respectively [11]. Overall, around 900 new cases of CE are expected in Italy every year [12].

In contrast, *E. multilocularis* has mainly been detected in Asia, Japan, China, North America, Central, and Eastern Europe [5,13]. There has been no evidence of its presence in the Mediterranean areas until recently, when the first human case of AE in Italy was described in a report [14]. 

The life cycles of both parasites involve two mammalian host species [15,16]. The definitive host is represented by carnivores, mainly dogs for *E. granulosus s.l.* and foxes or other wild canids and dogs for *E. multilocularis*, which harbor the adult stages in the intestine and release eggs through feces into the environment. The intermediate host is typically represented by ungulates for *E. granulosus s.l.*, and by rodents for *E. multilocularis*. Humans are dead-end hosts as they play no role in maintaining the life cycles of the parasites [17,18]. 

If embryonated eggs are ingested with contaminated food, they hatch in the small intestine and release a larval stage, known as the oncosphere. After penetrating the mucosa, the oncosphere travels via the bloodstream or the lymphatic system; in most cases it reaches the liver; followed by the lungs; and, less frequently, other sites like the spleen, kidneys, heart, bones, and central nervous system [19]. In the organs, the oncosphere further develops into the metacestode stage, which slowly grows, forming a cyst-like parasitic structure for *E. granulosus s.l.* and a tumor-like tissue mass for *E. multilocularis* [15,20]. 

The taxonomic subdivision of *E. granulosus s.l.* comprises five species: *E. granulosus sensu stricto* (*s.s.;* G1 and G3 genotype), *E. ortleppi* (G4), *E. equinus* (G5), *E. canadensis* (G6–G8 and G10), and *E. felidis* [21,22]. On the other hand, several studies have reported little variance among *E. multilocularis* genotypes [23].

CE diagnosis [24] in humans is mainly performed using imaging techniques (ultrasound, conventional radiography, magnetic resonance, and computed tomography), which are very useful tools [25,26]. However, they frequently need to be supported by other examinations, in particular in the case of differential diagnosis [27], neoplasia [7], or abscess [28]. Serological tests (enzyme linked immunosorbent assay (ELISA) and immunochromatographic test (ICT), along with immunoblotting (IB)) [29,30] are able to detect IgG antibodies directed against *E. granulosus s.l.* and *E. multilocularis*, and represent a valid tool to support doubtful radiological exams. However, these serological tests are often limited by cross-reactivity with other helminthic diseases, especially in the case of CE assays [31]. Moreover, in early and/or late stage cysts, a low performance has been reported [32]. However, *in vivo* investigations may be supported by further analyses performed directly on the cyst after surgical enucleation. Molecular analysis along with histopathology are the most reliable techniques to confirm the disease [26]. While molecular analysis is useful to differentiate species and genotypes following DNA analysis, histopathology presents a high specificity for recognizing the typical feature of the parasitic tissue, for example using hematoxylin–eosin (H/E) staining or immunohistochemistry (IHC), to directly detect the zoonotic agent using monoclonal antibodies (mAbs). The molecular analysis has to be performed on fresh or promptly frozen biological material. Indeed, protocols of DNA extraction on paraffin fixed formalin embed (PFFE) tissues often fail due to degraded genomic material in the presence of formalin [33]. Consequently, molecular analysis has several limits, such as having to perform retrospective surveys on stored PFFE samples [34]. 

Conversely, IHC presents several advantages for the direct detection of the parasite for both diagnostic and research purposes. Moreover, IHC is able to distinguish between *E. granulosus s.l.* and *E. multilocularis* using two different mAbs [35]. In detail, mAbEm2G11 is directed against the mucin-type Em2-glycoprotein specific for *E. multilocularis* [36], while mAbEmG3 is directed against an *Echinococcus* spp. specific antigen that has not been characterized yet [35,37]. Compared with molecular analysis, a dual staining approach using these two mAbs has another advantage. In the case of degenerated lesions and degraded DNA, where PCR fails, IHC represents a more sensitive alternative [35]. 

The main objective of this research was to test and investigate the reliability of a diagnostic tool in a small number of samples, which had previously been set up with a big sample size [35]. For this purpose, a survey was performed on samples of *E. granulosus s.l.* that originated from Sardinia. In detail, in this study, we wanted to test an IHC protocol with two monoclonal antibodies (mAbEmG3 and mAbEm2G11) able to confirm the clinical diagnosis of CE in Sardinia. In particular, in those cases in which radiological techniques and immunological analyses usually failto provide a correct or conclusive diagnostic answer. An early and prompt identification of CE allows for an adequate medical treatment and the correct follow-up of patients. In addition, the data obtained from this survey aimed to provide important information from an epidemiological point of view and may contribute to filling the gap that still exists regarding Echinococcosis for a specific geographical area, such as Sardinia.

## 2. Materials and Methods

### 2.1. Patients

A total of 13 patients were included in this retrospective study. These subjects referred to different Sardinian Hospitals (Italy) from 2017 to 2022 with symptoms compatible with CE. Several investigations on these patients and their samples have already been performed and described [38]. The previously published information on the 13 studied samples can be summarized as follows: clinical and laboratory analyses were carried out using imaging techniques, serological analysis, parasitological examination, and molecular characterization. A cystic lesion was evidenced in all subjects, but 12 specimens presented the pathognomonic signs of an echinococcal cyst: an oval or round shape lesion, laminated and germinal layers, and the presence of daughter cysts and fluid. Moreover, microscopic investigation evidenced the presence of protoscoleces in 11 cystic liquid samples, confirming their fertile status. Finally, the Sanger sequencing of mtgenes *cox1* and *nad5* of the successfully amplified fragments of these 11 samples led to clearly distinguishing between G1 (*n* = 9) and G3 (*n* = 2) genotypes of *E. granulosus s.l.* (Table 1).

These previous data [38] were very useful as a comparison with those obtained in this study and were used to confirm the new findings (displayed in Table 1).

### 2.2. Histopathological Analysis

An aliquot of each parasitic sample, collected during previous examination, was promptly fixed in 10% formalin and embedded in paraffin, following routine appropriate laboratory methods. Briefly, sections of 3–4 µm were serially cut from paraffin blocks, and the slices were collected on glass slides in a thermostatic bath and then subjected to two different protocols using an automatic stainer. To highlight the nucleus and cytoplasm of the cells, H/E staining was performed. Subsequently, to determine the parasitic source of the cyst in question, an IHC procedure [39] was used by two different mAbs, mAbEmG3 and mAbEm2G11, which were able to identify *Echinococcus* spp. and *E. multilocularis* [35], respectively. The monoclonal antibodies were produced *in vitro* from murine cell lines, as already described [36], and are available upon request (peter.deplazes@uzh.ch). Moreover, the IHC method required that the tissue slice was placed on a positively charged glass slide.

## 3. Results

### 3.1. Patients

The 13 patients involved in the study comprised 8 males and 5 females—their age ranged between 18 and 78 years with a mean of 51.5 (standard deviation of ±18.8). Three patients, although residents of Italy, came from other countries, such as Romania, Morocco, and Ghana. 

### 3.2. Histopathological Analysis

**Figure 1:**
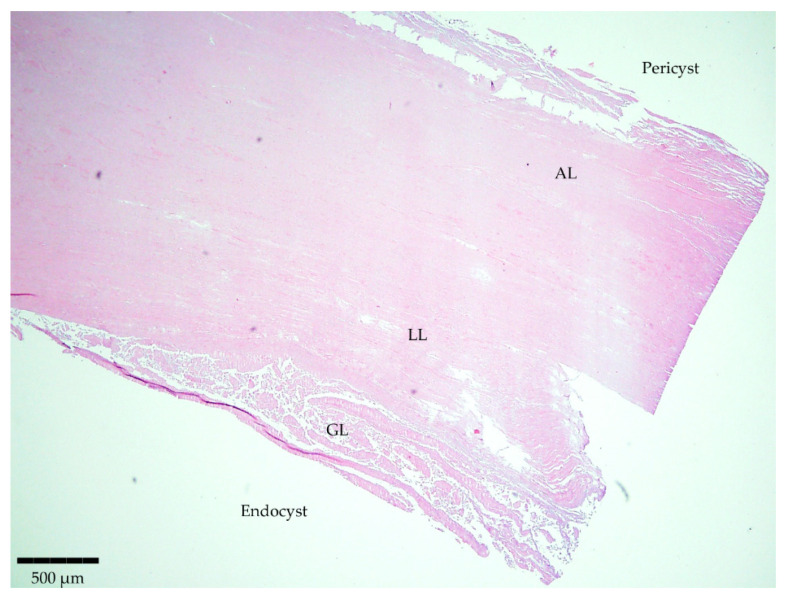
Microscopic observation of a histopathological section stained with H/E evidencing the typical parasite features. Magnitude 4×; scale bar: 500 µm. Legend: adventitial layer (AL), laminated layer (LL), and germinal layer (GL).

Staining of the sliced samples with H/E evidenced the typical parasite features for 12 samples out of the 13 involved in the study (Table 1). The structure was characterized by separate layers (Figure 1). Firstly, the host-produced granulomatous reaction surrounded all parasitic cystic structures with an adventitial layer (AL), besides one thick acellular and laminated layer (LL) and a cellular germinal layer (GL). Finally, brood capsules with protoscoleces could be observed in fertile cysts. Only one histological section was considered negative as it showed non-parasitic characteristics.

**Figure 2:**
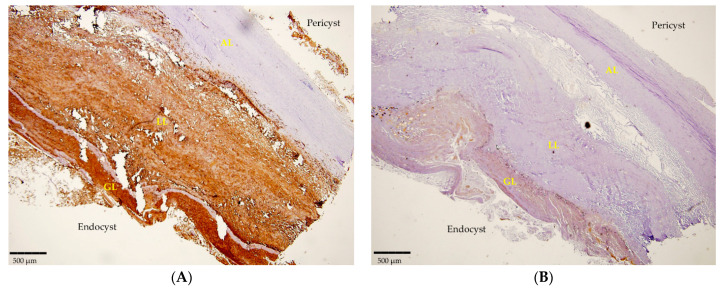
(**A**) Section of a cyst positive for *E. granulosus s.s.* detected by IHC using mAbEmG3. (**B**). Negative IHC section of *E. granulosus s.s.* cyst incubated with mAbEm2G11. Magnitude 4×; scale bar: 500 µm. Legend: adventitial layer (AL), laminated layer (LL), germinal layer (GL).

Likewise, 12 histological sections that were examined clearly revealed the positivity detected by mAbs in IHC. The mAbEmG3 displayed and confirmed the specificity for *E. granulosus s.s.* samples, evidenced by the typical brownish color of the reactive layers of the GL, comprising protoscoleces, and LL, visible using optical microscopy (Figure 2A). Furthermore, small antigenic particles of *E. granulosus* (SPEGS) were often detected outside of the laminated layer in the host tissue surrounding the parasite lesions. One negative slide was also evidenced. In addition, after incubation with mAbEm2G11, all preparations were negative (Figure 2B). 

## 4. Discussion

The diagnosis of CE is considered challenging as cases can be asymptomatic for years and there are no pathognomonic signs of the disease until the parasitic lesion reaches a considerable size; Sometimes, cyst formation can be confused with another disorder; thus, it becomes necessary to perform a differential diagnosis. 

This survey aimed to test the reliability of a diagnostic tool, previously set up [35], on samples of *E. granulosus s.l.* originated from Sardinia, as, to our knowledge, the IHC protocol with two monoclonal antibodies (mAbEmG3 and mAbEm2G11) has never been used on a Sardinian strain. However, the three patients who were not born in Sardinia but were residents at the time of diagnosis lacked further data to confirm the origin of the infection.

IHC is useful to support the analytical process for providing a clear diagnostic picture of CE. In detail, in this study, we wanted a test able to confirm the clinical diagnosis of CE in Sardinia; in particular in those cases in which radiological techniques and immunological analyses failed to provide a correct or conclusive diagnostic answer. Early and prompt identification of CE allows for adequate medical treatment and the correct follow-up of patients. In addition, data obtained from this survey aimed to provide important information from an epidemiological point of view, and may contribute to filling the gap in knowledge regarding Echinococcosis in a specific geographical area, such as Sardinia. For this reason, 13 patients suspected to harbor an echinococcal cyst after being abdominally examined using imaging techniques were involved in this study. 

Despite radiological techniques being considered the most reliable exams for CE detection [26,32], sometimes, they fail to perform a correct diagnosis of CE. Indeed, other pathologies such as abscess or neoplasia could be wrongly detected and confused with an hydatid cyst; in particular, if the inner biologic material is liquid or even lacking [7]. It has been reported that hepatic CE and pulmonary cysts can radiologically look like malignant or infectious diseases such as neoplasia or tuberculosis [40]. 

Immunological analyses support radiological tools by providing a correct and conclusive diagnostic response; however, they occasionally lack sensitivity and specificity [29,31,41] in particular for cases of early (CE1) or late stage (CE4/5) CE and cross-reaction for other parasitosis [32]. 

Noticeably, improved detection of the CE etiological agent has been found through investigations performed directly on parasitological material using PCR and sequencing. All of the detected isolates were previously characterized as *E. granulosus s.s*. and resulted in G1 (*n* = 9) and G3 (*n* = 2) genotypes, confirming the presence of a higher concentration of G1 in Sardinia [38]. 

The histopathological analysis was able to shed light on this complex diagnostic picture (Table 1). In this study, a total of 12 samples were positivite for CE; in contrast, 1 sample was diagnosed as being of a non-parasitological origin, as it resulted in a neoplasia. The distinctive characteristic structure of an *Echinococcus* cyst was shown by H/E, while a positive reaction by IHC was evidenced by mAbEmG3 (Figure 2A). As reported in a previous study [35], a high sensitivity and specificity were reported for this newly established mAb as it was able to detect all CE samples demonstrating 100% positivity and was of capable of identifying PCR negative samples that were stage CE5 and sterile. Moreover, the sample of a non-parasitological source was confirmed negative. Furthermore, mAbEmG3 was able to identify *E. granulosus s.l.* in only one step, avoiding amplification and sequencing protocols. Further, as established by other studies [35,36,42], after incubation with mAbEm2G11, all preparations were negative (Figure 2B), confirming the affinity and specificity of this antibody to *E. multilocularis*.

Hence, to have a consistent and conclusive diagnosis, a multidisciplinary approach is required, and the contribution of different techniques is the only way to guarantee the correct identification of this zoonosis. A conclusive and early diagnosis of CE in human patients is often essential so that correct clinical and pharmacological management and follow up of the patient can be performed [26]. Moreover, several accurate laboratory and clinical investigations in the direction of a differential diagnosis need to be performed during these examinations [27]. The need for a reliable diagnostic tool becomes necessary, in particular for unclear and difficult cases. 

Moreover, we believe the present findings improve the knowledge on histopathology in the scientific and diagnostic field of echinococcosis. In this study, we also wanted to test the newly established mAbs on *E. granulosus s.s.* strains with a Sardinian origin. Even if Sardinia, similar to other Mediterranean areas, has only been characterized by the presence of *E. granulosus s.l.* [38], the need to develop techniques able to discriminate between CE and AE [43] is of vast importance in several geographic zones worldwide [44]; in particular, because of the recent findings of an autochthonous case of human AE in Italy [14].

## 5. Conclusions

Our findings confirm that IHC, by means of mAbEmG3 and mAbEm2G11, is a reliable diagnostic tool to confirm the diagnosis of CE in human patients. As a very high performance was presented upon detection of the *E. granulosus s.l.* Sardinian strain, IHC is particularly useful in those cases in which other techniques fail to provide a correct or conclusive diagnosis. 

## Figures and Tables

**Table 1:** Findings on clinical and laboratory investigations previously published (*) [38] compared with the immunohistochemistry results of 13 patients suspected of CE and their cystic samples.

Cyst/ Patients	Stage *	Serology * ELISA/IB	Molecular Analysis		Parasitological Examination *	Histology * H/E	Immunohistochemistry	
			PCR cox1/nad5*	Genotyping *			MabEm2G11	MabEmG3
1	CE2	positive	P	G1	parasitic features	P	N	P
2	CE2	positive	P	G1	parasitic features	P	N	P
3	CE2	positive	P	G1	parasitic features	P	N	P
4	CE2	positive	P	G3	parasitic features	P	N	P
5	CE3b	positive	P	G1	parasitic features	P	N	P
6	CE3b	positive	P	G1	parasitic features	P	N	P
7	CE3b	positive	P	G3	parasitic features	P	N	P
8	CE3b	positive	P	G1	parasitic features	P	N	P
9	CE3b	positive	P	G1	parasitic features	P	N	P
10	N.D.	positive	P	G1	parasitic features	P	N	P
11	CE4	positive	P	G1	parasitic features	P	N	P
12	CE5	positive	N	N.D.	parasitic features	P	N	P
13	CE3b	negative	N	N.D.	non-parasitic source	N	N	N

P = positive; N = negative.

## Data Availability

The authors are available to share data related to the manuscript; no further data were created or analyzed in this study.
